# Ants are more than just curious bystanders to some flowers—they act as significant pollinators

**DOI:** 10.3389/finsc.2023.1145761

**Published:** 2023-06-15

**Authors:** Susmita Das, Amlan Das

**Affiliations:** Entomology Laboratory, Department of Zoology, University of Calcutta, Kolkata, West Bengal, India

**Keywords:** ant pollination, myrmecophilous plant, floral characteristics, pollination network, seasonal bias

## Abstract

Ant–plant associations are ubiquitous and highly diverse in almost all terrestrial environments, resulting in complex ecological networks. Although ant–plant mutualism is prevalent, ant-mediated pollination is uncommon, and only a few investigations have demonstrated their role in pollination. Thus, the topic of ant-mediated pollination requires revision to assess its significance in pollination biology. Ants are frequent floral visitors, but their impact on plant reproductive fitness is rarely acknowledged; nonetheless, numerous flower-visiting ants have been investigated for their involvement in promoting floral development and hybrid vigor in crops. In this study, we present a summary of the scientific literature published over the last four decades on ants’ involvement in pollination, the diversity of pollinating ants to various host plants, the ant–plant pollinating networks, and seasonal patterns of ant-mediated pollination. Ants generally forage for flowers in quest of nectar and other sustenance, and in doing so they pollinate the flowers that they encounter. This review identified the pollination networks between ants and plants at the species and family levels. Pollination is often affected by a number of aspects, including the flower’s sex, its ovary position, the inflorescence it bears, and the time of year. The available literature demonstrates that ants visit the inflorescences of the same species only to promote cross-pollination, a process known as “geitonogamy”; however, we conclude that ants may visit different inflorescences of different plants in the field. If ant pollination is the norm, there is less selection pressure to acquire self-compatibility; nonetheless, ants' cross-pollination may have caused ants to co-evolve with the pollinating flowers. This indicates that ants are more than just curious bystanders to some flowers; they act as significant pollinators.

## Introduction

Nature’s beauty and grace are sleeping into the seed vessels, which is only possible through the diverse pollination strategies in plants and insects under self and cross-pollination modes (1). A floral visitor’s efficacy can be measured in terms of its contribution to plant health (2–4); similarly, the visitor’s pollination performance is also correlated to its fitness (5). The concept of the co-evolution of flowering plants and their pollinators (6, 7) originated many decades ago (8) on the basis of numerous remarkable interactions between flowers and insects that transfer pollen to floral stigma (9, 10). Flower–insect co-adaptation is an ongoing process in which the partnership is developed by reducing reproductive obstacles through pollen transfer (11). Although entomophily pollinators are diverse (12), ant-mediated pollination is less prevalent than that of other pollinating species (13). It has been reported that ants have a limited pollination ability (13). However, there might be other reasons for the lack of research on the subject; had the scenario been reversed, the probabilities would have differed.

A well-organized mutualistic relationship develops when ants engage with plants to transfer pollen (14). Plants defend against herbivores; sometimes, it may be costly to exclude mutualists, as seen in aggressive bodyguard ants, which repel hovering pollinators (15). As a result, resource allocation might cause trade-offs between plant defense and reproductive success (16). A plant’s interactions with other species may also provide a “context” for defining its costs. For example, foliar herbivory can influence floral traits (17), and natural enemies can exert selection pressure that runs counter to pollinators’ “labor” (18, 19). Plant defense can thus be accompanied by reduced competitive ability or the “deterrence” of mutualists (20). Their structures (21, 22), scents (23), and phenologies (24) aid plants in repelling natural enemies by limiting pollinator access. Bodyguard ants can indirectly defend plants by attacking growing blossoms (25, 26). Such interactions may limit conflicts between bodyguard ants and pollinators (27, 28); therefore, bodyguard ants provide opportunities to explore conflicts between plant defense and reproduction (15).

Since the frequency of ants’ visits to myrmecophilous plants is dependent on the plant’s health (29, 30), angiosperms have evolved different flower arrangements, with brilliant colors, perfumes, and enhanced nectar production. Like many other entomo-pollinators, ants are drawn to angiosperms because of their rich morphological textures and modifications (31). In most instances, ant-mediated pollination is supposed to be a low-energy system; while not using energetically costly resources to attract insect pollinators, plants have evolved specific traits for attracting ants (32). Ants as pollination vectors may be favored in habitats where they are abundant and where local vegetation supports open flowers with ground-level inflorescences (33–35). However, the pollination ability of ants can be hindered by multiple factors, including their tendency to self-groom and their small body size compared with the reproductive structures of the flowers they encounter (36). Furthermore, pollen germination may be inhibited by their metapleural gland secretions (37, 38), which may not favor pollination. Despite ants’ preference for nectar, which facilitates floral visitation, they can sometimes impede pollination by destroying flowers (39), feeding on pollen (40–42), or interfering with or repelling other pollinating vectors (43–46).

Traditional ant–plant network analysis has focused on bipartite grids that primarily address a single node of ecological interaction, such as “ant–flower,” “ant–seed,” or “ant–nectar” (47, 48). However, in ecological communities, ant–plant interactions can be multifaceted. One species of ant may interact with various plants and fulfill multiple roles, such as pollinator, disperser, protector, and neuter (49, 50). Therefore, ant–plant interactions in a particular ecosystem can be incorporated into a multi-network species interaction in a multi-trophic environment, allowing their affiliations to be quantified as a significant model for “biodiversity interaction” (50–52). These networks play a crucial role in determining the “interaction diversity” of many ecosystems, particularly those in the tropics (52–54). Therefore, their prevalence makes them one of the most significant faunas on earth (55). Ants’ diets vary according to species; some are predators, while others feed on seeds, honey, or fungus (56). They occupy most terrestrial environments, and constitute 10%–15% of the total animal biomass. The dominance of angiosperms in most terrestrial ecosystems permits various facultative and obligatory connections between plants and insects. Aside from ants’ floral nectar-gathering behavior, their visiting of extra-floral nectaries forms part of plants’ indirect anti-herbivory defense strategy since ants often attack predatory arthropods while patrolling and thus protect the plants.

Although insect–plant interactions have been extensively researched, the role of ants as pollinators has received remarkably less attention. Ant pollination is an unusual mutualistic interaction (57), yet their pollination links with plants are often defined as reciprocal mutualism. Such pollinating relationships are often regarded as hostile since they are likely to fight or interact with other heterospecific pollinators (53). However, a significant amount of mutual exchange of resources may occur through their pollination skills. The primary goals of this review are to examine the diversity of pollinating ants and recognize their potential hosts where they actively pollinate. Since plant–herbivore networks often demonstrate how insects interact with plants in specialist or generalist capacities, this review concentrates on understanding the potential efficacy of ants’ pollination performance and efficiency. Interacting networks among ants, as pollinators, for a specific or broad range of plant communities have also been investigated.

## Materials and methods

### Data collection

The current study investigates three key concerns and affinities between pollinating ants and the plant communities they encounter: (a) pollinating ant diversity (species and subfamilies), plant diversity (species and families), and the ant–plant network. (b) the importance of floral features (inflorescence, sexuality, and ovarian style) in pollination, and (c) pollination phenology.

### Data acquisition

#### Data sampling

More than 100 peer-reviewed published relevant studies from the last four decades (1981–2022) were analyzed during the review. We collected data only from studies in which authors reported on “true pollination”; those which contained references to observations of “flower visits” without a depiction of “true pollination” were ignored. There were three main categories of information in the available literature:

1. An explicit mention of ant species to plant species where ant-mediated pollination was documented.
2. An explicit mention of ant-mediated pollination without stating the names of the ant or plant species.
3. An indefinite, probabilistic view of ant pollination on some plants (or ant–plant interactions in general) where both the interacting species names (ant and plant) were either mentioned or absent.

Therefore, we narrowed the data to only the most relevant and clear examples of pollination at the species and genus levels. The data used to create the visual depictions of ant–plant networks and connections were pulled from over 50 articles (**Table 1**). The bipartite graphs were created with R-Studio software, although additional supporting data were obtained from other scholarly works. In the literature review, studies related to ants of varying subfamilies pollinating plants across all species and families to varying degrees were gathered. The pollinated flowers’ inflorescence, floral sexuality (unisexual or bisexual), and ovarian styles (epigynous, hypogynous, or perigynous) were all documented.

**Table 1:** Diversity of pollinating ants with respect to pollinated plants, floral characteristics, pollinating season, and study sites.

Pollinating ant		Pollinating plant		Flower characteristics			Pollinatating season		Observed location	Reference
Name	Family	Name	Family	Inflorescence	Sex	Ovary position	As described in literature	After conversion		
*Anoploilepis gracilipes*	Formicinae	*Salacia oblonga*	Celastraceae	Racemose	Bisexual	Hypogynous	January to July	Winter, Suimmer, Rainy season	Peechi, Kerala, India	(58)
"	"	*Salacia gambleana*	"	"	"	"	January to June	"	"	(58)
*Brachymyrmex termitophilus*	"	*Blutaparon portulacoides*	Amaranthaceae	Capitulum	"	"	October to April/May to September	Rainy season, Winter	São Paulo, Brazil	(32)
*Camponotus compressus*	"	*Cucurbita moschata*	Cucurbitaceae	Solitary	Unisexual	Epigynous	Winter, Summer	Winter, Summer	Gazipur, Bangladesh	(59)
*Camponotus crassus*	"	*Cocos nucifera*	Arecaceae	Racemose	"	Hypogynous	November, December, January	Rainy season	Bahia, Brazil	(60)
"	"	*Paepalanthus lundii*	Eriocaulaceae	Capitulum	"	"	October, November, December	"	Minas Gerais, Brazil	(36)
*Camponotus foreli*	"	*Frankenia thymifolia*	Frankeniaceae	Racemose	"	"	Summer	Summer	Granada, Spain	(34)
"	"	*Retama sphaerocarpa*	Fabaceae	Panicle	Bisexual	"	"	"	"	(34)
*Camponotus japonicus*	"	*Epipactis thunbergii*	Orchidaceae	Racemose	"	Epigynous	Rainy Season	Rainy season	Kumamoto, Japan	(61)
*Camponotus micans*	"	*Lobularia maritima*	Brassicaceae	"	"	Hypogynous	Spring, Summer, Autumn/Winter	Spring, Summer, Autumn/Winter	Cádiz, Spain	(62)
*Camponotus molossus*	"	*Conospermum undulatum*	Proteaceae	Capitulum	Unisexual	"	August, September, October	Winter, Spring	Swan Coastal Plain, Australia	(57)
*Camponotus parius*	"	*Jatropha curcas*	Euphorbiaceae	Cymose	"	"	April, May, June	Spring, Summer	Jinsha Valley, China	(63)
*Camponotus pilicornis*	"	*Cytinus hypocistis*	Cytinaceae	Racemose	"	Epigynous	All over the year	Summer, Winter, Spring, Autumn	Huelva, Spain	(35)
*Camponotus ruber*	"	*Naufraga balearica*	Apiaceae	"	Bisexual	"	April-August	Summer, Spring	Cap de Catalunya, Spain	(64)
*Camponotus sp*	"	*Mangifera indica*	Anacardiaceae	Panicle	"	Hypogynous	January, February, March	Winter, Spring	Perlis, Malaysia	(65)
"	"	*Frankenia thymifolia*	Frankeniaceae	Racemose	Unisexual	"	Summer	Summer	Granada, Spain	(34)
*Camponotus terebrans*	"	*Conospermum undulatum*	Proteaceae	Capitulum	"	"	August, September, October	Winter, Spring	Swan Coastal Plain, Australia	(57)
*Formica cunicularia*	"	*Euphorbia cyparissias*	Euphorbiaceae	Cyathium	"	"	April	Spring	Swiss Jura mountains, Switzerland	(66)
"	"	*Euphorbia seguieriana *	"	"	"	"	August	Summer	Würzburg, Germany	(67)
*Formica fusca*	"	*Trinia glauca*	Apiaceae	Racemose	Bisexual	Epigynous	May to September	Summer, Autumn, Spring	Avon Gorge, England	(68)
*Formica lemani*	"	*Chamorchis alpina*	Orchidaceae	"	"	"	July	Summer	Cadagno, Switzerland	(69)
*Formica neorufibarbis*	"	*Paronychia pulvinata*	Caryophyllaceae	Solitary	"	Hypogynous	Summer	"	Colorado, USA	(70)
*Formica polyctena*	"	*Epipactis palustris*	Orchidaceae	Racemose	"	Epigynous	July	"	Groningen, Netherlands	(71)
*Formica pratensis*	"	*Euphorbia cyparissias*	Euphorbiaceae	Cyathium	Unisexual	Hypogynous	April	Spring	Swiss Jura mountains, Switzerland	(66)
*Formica rubra*	"	*Cucurbita moschata*	Cucurbitaceae	Solitary	"	Epigynous	Winter, Summer	Winter, Summer	Gazipur, Bangladesh	(59)
*Formica subsericea*	"	*Fragaria virginiana*	Rosaceae	Cymose	Bisexual	Hypogynous	May	Spring	Pennsylvania, USA	(72)
*Formica schaufussi*	"	*Diamorpha smallii*	Crassulaceae	"	"	"	March	"	Rutherford, Carolina	(73)
*Lasius alienus*	"	*Euphorbia cyparissias*	Euphorbiaceae	Cyathium	Unisexual	"	April	"	Swiss Jura mountains, Switzerland	(66)
"	"	*Trinia glauca*	Apiaceae	Racemose	Bisexual	Epigynous	May to September	Summer, Autumn, Spring	Avon Gorge, England	(68)
*Lasius cinereus*	"	*Borderea chouardii*	Dioscoreaceae	"	Unisexual	"	_	_	_	(74)
*Lasius grandis*	"	*Apium bermejoi*	Apiaceae	"	Bisexual	"	April-August	Summer, Spring	Menorca, Spain	(75)
"	"	*Borderea chouardii*	Dioscoreaceae	"	Unisexual	"	_	_	_	(74)
"	"	*Naufraga balearica*	Apiaceae	"	Bisexual	"	April-August	Summer, Spring	Cap de Catalunya, Spain	(64)
*Lasius niger*	"	*Epipactis palustris*	Orchidaceae	"	"	"	July	Summer	Groningen, Netherlands	(71)
*Oecophylla smaragdina*	"	*Salacia gambleana*	Celastraceae	"	"	Hypogynous	January to June	Winter, Suimmer, Rainy season	Peechi, Kerala, India	(58)
"	"	*Salacia oblonga*	"	"	"	"	January to July	"	"	(58)
*Paratrechina flavipes*	"	*Balanophora tobiracola*	Balanophoraceae	"	Unisexual	Epigynous	November, December, January	Autumn, Winter	Kagoshima, Japan	(76)
*Paratrechina vividula*	"	*Jatropha curcas*	Euphorbiaceae	Cymose	"	Hypogynous	April, May, June	Spring, Summer	Jinsha Valley, China	(63)
*Prenolepis imparis*	"	*Epifagus virginiana*	Orobanchaceae	Solitary	Bisexual	"	September	Autumn	Piedmont, Coastal Plain, USA	(77)
"	"	*Fragaria virginiana*	Rosaceae	Cymose	"	"	May	Spring	Pennsylvania, USA	(72)
*Proformica longiseta*	"	*Alyssum purpureum*	Brassicaceae	Racemose	"	"	Summer	Summer	Granada, Spain	(34)
"	"	*Arenaria tetraquetra*	Caryophyllaceae	Cymose	"	"	"	"	"	(34)
"	"	*Hormathophylla spinosa*	Brassicaceae	Racemose	"	"	June, July, August	"	Sierra Nevada, Spain	(78)
"	"	*Sedum anglicum*	Crassulaceae	Cymose	"	"	Summer	"	Granada, Spain	(34)
*Plagiolepis pygmaea*	"	*Apium bermejoi*	Apiaceae	Racemose	"	Epigynous	April-August	Summer, Spring	Menorca, Spain	(75)
"	"	*Cytinus hypocistis*	Cytinaceae	"	Unisexual	"	All over the year	Summer, Winter, Spring, Autumn	Huelva, Spain	(35)
"	"	*Naufraga balearica*	Apiaceae	"	Bisexual	"	April-August	Summer, Spring	Cap de Catalunya, Spain	(64)
*Plagiolepis schmitzii*	"	*Cytinus hypocistis*	Cytinaceae	"	Unisexual	"	All over the year	Summer, Winter, Spring, Autumn	Huelva, Spain	(35)
*Plagiolepis wroughtoni*	"	*Jatropha curcas*	Euphorbiaceae	Cymose	"	Hypogynous	April, May, June	Spring, Summer	Jinsha Valley, China	(63)
*Aphaenogaster senilis*	Myrmicinae	*Cytinus hypocistis*	Cytinaceae	Racemose	Unisexual	Epigynous	All over the year	Summer, Winter, Spring, Autumn	Huelva, Spain	(35)
*Aphaenogaster sp*	*"*	*Balanophora tobiracola*	Balanophoraceae	"	"	"	November, December, January	Autumn, Winter	Kagoshima, Japan	(76)
*Crematogaster auberti*	*"*	*Cytinus hypocistis*	Cytinaceae	"	"	"	All over the year	Summer, Winter, Spring, Autumn	Huelva, Spain	(35)
*Crematogaster politula*	*"*	*Jatropha curcas*	Euphorbiaceae	Cymose	"	Hypogynous	April, May, June	Spring, Summer	Jinsha Valley, China	(63)
*Crematogaster scutellaris*	*"*	*Cytinus hypocistis*	Cytinaceae	Racemose	"	Epigynous	All over the year	Summer, Winter, Spring, Autumn	Huelva, Spain	(35)
*Crematogaster sp*	*"*	*Epifagus virginiana*	Orobanchaceae	Solitary	Bisexual	Hypogynous	September	Autumn	Piedmont, Coastal Plain, USA	(77)
*Leptothorax acervorum*	*"*	*Chamorchis alpina*	Orchidaceae	Racemose	"	Epigynous	July	Summer	Cadagno, Switzerland	(69)
*Leptothorax tuberum*	*"*	*Borderea pyrenaica*	Dioscoreaceae	"	Unisexual	Hypogynous	June	"	Pineta Valley, Spain	(79)
"	*"*	*"*	"	"	"	"	_	_	Huesca, Saravillo, Spain	(80)
*Leptothorax sp*	*"*	*Balanophora kuroiwai*	Balanophoraceae	"	"	Epigynous	January to April, December	Winter, Spring	Okinawa, Japan	(76)
*Leptothorax fuentei*	*"*	*Frankenia thymifolia*	Frankeniaceae	"	"	Hypogynous	Summer	Summer	Granada, Spain	(34)
*Monomorium pharaonis*	*"*	*Cucumis sativus*	Cucurbitaceae	Solitary	"	Epigynous	From May to two years	Rainy season, Winter	Obio-Akpor, Nigeria	(81)
*Myrmica ruginodis*	*"*	*Cardiocrinum cordatum*	Liliaceae	Racemose	Bisexual	Hypogynous	July	Summer	Hokkaido Obihiro, Japan	(82)
*Myrmecia urens*	*"*	*Leporella fimbriata*	Orchidaceae	"	"	Epigynous	_	_	_	(83)
*Pheidole pallidula*	*"*	*Apium bermejoi*	Apiaceae	"	"	"	April-August	Summer, Spring	Menorca, Spain	(75)
"	*"*	*Cytinus hypocistis*	Cytinaceae	"	Unisexual	"	All over the year	Summer, Winter, Spring, Autumn	Huelva, Spain	(35)
"	*"*	*Naufraga balearica*	Apiaceae	"	Bisexual	"	April-August	Summer, Spring	Cap de Catalunya, Spain	(64)
*Pheidole reichenspergeri*	*"*	*Blutaparon portulacoides*	Amaranthaceae	Capitulum	"	Hypogynous	October to April/May to September	Rainy season, Winter	São Paulo, Brazil	(32)
*Solenopsis sp*	*"*	*Mangifera indica*	Anacardiaceae	Panicle	"	"	Spring	Spring	Abo-Hammad, Sharkia, Egypt	(84)
*Tetramorium ruginode*	*"*	*Cytinus hypocistis*	Cytinaceae	Racemose	Unisexual	Epigynous	All over the year	Summer, Winter, Spring, Autumn	Huelva, Spain	(35)
*Tetramorium semilaeve*	*"*	*"*	"	"	"	"	"	"	"	(35)
*Dorymyrmex nigra*	Dolichoderinae	*Blutaparon portulacoides*	Amaranthaceae	Capitulum	Bisexual	Hypogynous	October to April/May to September	Rainy season, Winter	São Paulo, Brazil	(32)
*Iridomyrmex anceps*	*"*	*Jatropha curcas*	Euphorbiaceae	Cymose	Unisexual	"	April, May, June	Spring, Summer	Jinsha Valley, China	(63)
*Iridomyrmex gracilis*	*"*	*Microtis parviflora*	Orchidaceae	Racemose	Bisexual	Epigynous	_	_	Sydney, Australia	(85)
*Iridomyrmex purpureus*	*"*	*Conospermum undulatum*	Proteaceae	Capitulum	Unisexual	Hypogynous	August, September, October	Winter, Spring	Swan Coastal Plain, Australia	(57)
*Iridomyrmex sp*	*"*	*Blandfordia grandiflora*	Blandfordiaceae	Racemose	Bisexual	"	January	Summer	New South Wales, Australia	(86)
*Tapinoma erraticum*	*"*	*Euphorbia seguieriana *	Euphorbiaceae	Cyathium	Unisexual	"	August	"	Würzburg, Germany	(67)
*Tapinoma madeirense*	*"*	*Apium bermejoi*	Apiaceae	Racemose	Bisexual	Epigynous	April-August	Summer, Spring	Menorca, Spain	(75)
*Tapinoma melanocephalum*	*"*	*Jatropha curcas*	Euphorbiaceae	Cymose	Unisexual	Hypogynous	April, May, June	Spring, Summer	Jinsha Valley, China	(63)
*Tapinoma nigerrimum*	*"*	*Arenaria tetraquetra*	Caryophyllaceae	"	Bisexual	"	Summer	Summer	Granada, Spain	(34)
"	*"*	*Cytinus hypocistis*	Cytinaceae	Racemose	Unisexual	Epigynous	All over the year	Summer, Winter, Spring, Autumn	Huelva, Spain	(35)
*Tapinoma sessile*	*"*	*Fragaria virginiana*	Rosaceae	Cymose	Bisexual	Hypogynous	May	Spring	Pennsylvania, USA	(72)
*Technomyrmex albipes*	*"*	*Syzygium occidentale*	Myrtaceae	"	"	Epigynous	December, January, February	Winter	Kerala, India	(87)
*Temnothorax albipennis*	*"*	*Trinia glauca*	Apiaceae	Racemose	"	"	May to September	Summer, Autumn, Spring	Avon Gorge, England	(68)
*Temnothorax exilis*	*"*	*Neotinea maculata*	Orchidaceae	"	"	"	Spring	Spring	Valladolid, Spain	(88)
*Temnothorax recedens*	*"*	*Naufraga balearica*	Apiaceae	"	"	"	April-August	Summer, Spring	Cap de Catalunya, Spain	(64)
*Temnothorax sp*	*"*	*Chenorchis singchii*	Orchidaceae	"	"	"	February, March	Winter, Spring	Baoshan, Yunnan Province, China	(89)
*Pseudomyrmex gracilis*	Pseudomyrmecinae	*Cocos nucifera*	Arecaceae	Racemose	Unisexual	Hypogynous	November, December, January	Rainy season	Bahia, Brazil	(60)
*Pseudomyrmex termitarius*	*"*	*"*	"	"	"	"	"	"	"	(60)
*Tetraponera sp*	*"*	*Salacia oblonga*	Celastraceae	"	Bisexual	"	January to July	Winter, Suimmer, Rainy season	Peechi, Kerala, India	(58)
"	*"*	*Salacia gambleana*	"	"	"	"	January to June	"	"	(58)

### Data weightage

We noticed that the pollinating activity of certain ant species was reported on frequently, whereas that of other species was discussed only occasionally, and that some species were studied still less frequently and hence mentioned only on rare occasions. We assigned statistical weightage to pollinating ant species according to their frequency in independent publications and rated the ants in order of high to low pollinator. However, ant species described by the same author/author-group in multiple publications (i.e., the same ant species described by the same author in separate literature) were disregarded. Likewise, multiple studies on an ant species in different times by the same author/author-group were also ignored. This was in place to avoid erroneous weightage of occurrence frequency of a particular ant. Therefore, in such cases, the pollinating frequency of that particular ant species, as per the record of the author/author group, was one. If two or more authors documented pollination on the same ant species (regardless of whether the pollinating plant was the same or different), the species weightage was powered by two or more depending on the records of the same species by different authors. Given the lack of reliable data on the frequency with which ants execute pollination, we opted for this approach. Despite the drawbacks of this method, it provides reliable indications as to which ant species are frequent pollinators (i.e., higher occurrence frequency of the same ant species by multiple authors) or rare pollinators (i.e., lower occurrence frequency of the same ant species by different authors) (34).

### Data assortment

All documented pollination events were classified according to pollinating ants (species and subfamily), pollinated plants (species and family), floral features (sex, ovary position, and inflorescence), and seasonal prevalence. Myrmecophilous ants have been shown to have clear seasonal preferences, with evidence spanning from summer to spring vegetation. During data analysis, it was observed that pollination events were recorded in a mix of timeframes (month, season, and period) across the study areas (country locations). Hence, we converted the literature-cited timings (month, season, period) into five seasons (summer, rainy season, autumn, winter, and spring) based on the established meteorological season for the countries. For example, if pollination was observed in Brazil from October onwards, the period was converted into the rainy season (32) according to the Brazilian meteorological calendar. Similarly, if pollination was recorded in Nigeria from May onwards, it was converted to the rainy season (84) according to the Nigerian meteorological calendar. Pollination recorded from June onwards in India was also attributed to the rainy season. These conversions were undertaken to obtain a more global picture of pollination seasons to account for the fact that meteorological seasons do not always align with calendar months depending on a country’s geographical hemisphere. All conversions (month to season) were carried out using, where available, data from the respective country’s official meteorological websites. The data sets were entered into Microsoft Excel, compared with reference documents, and analyzed.

### Data analysis

All records regarding the pollinating activity of ants on their different plants were analyzed and compared. The arbitrary interaction of each ant species with its pollinated plant(s) was estimated using data acquired at random from various literature studies. Binary data were organized in a Microsoft Excel file based on the arrangement of ant species with pollination plant species. We also investigated the interactions between pollinating plant families and ant sub-families based on our observations, and these data were structured in a similar matrix format. Throughout our analysis, ant morphospecies were treated as distinct species. Two interaction networks (or matrices) were created separately: (a) at the species level and (b) at the higher taxonomic level (family and subfamily). Data obtained from the network analysis were classified into two broad categories (1): plants’ exclusivity to ants—(i) the pollination of a single plant species by multiple ant species, (ii) the pollination of a limited number of plant species by multiple ant species, (iii) the pollination of a single plant species by limited species of ants, and (iv) the pollination of a single plant species by a single ant species—and (2) ants’ exclusivity to plants—(v) the pollination of multiple plant species by a single ant species, (vi) the pollination of a limited number of plant species by a single ant species, and (vii) the pollination of multiple plant species by multiple ant species.

The network data interpretation, construction, and metric analyses were performed in R software (version 4.0.3), utilizing the “Bipartite”, “Vegan”, and “Tiff” packages for graphical representations (90, 91). The nestedness metric based on the overlap and decreasing fill (NODF) metric network-level nestedness analysis (92) was also performed in R software. The NODF values range from 0 (completely non–nested network) to 100 (completely nested network) (92, 93). During calculation, the “H2′-fun” function performed network-level specialization (H2′), which denotes an index of “complementarity specialization” for the complete bipartite network (94). The H2′ range also varied from 0 (no specialization) to 1 (complete specialization) (91, 92). All matrices in the network analysis were non-weighted and followed a binary pattern where “0” indicates “no-pollination” and “1” indicates “pollination”.

The χ^2^ tests were employed to examine differences in pollinating ant subfamilies, pollinated plant families, flower sex (uni- or bisexual), floral styles (epigynous or hypogynous), and floral inflorescence varieties. The variances were graphically depicted and validated at significance levels of p = 0.05.

## Result

**Figure 1:**
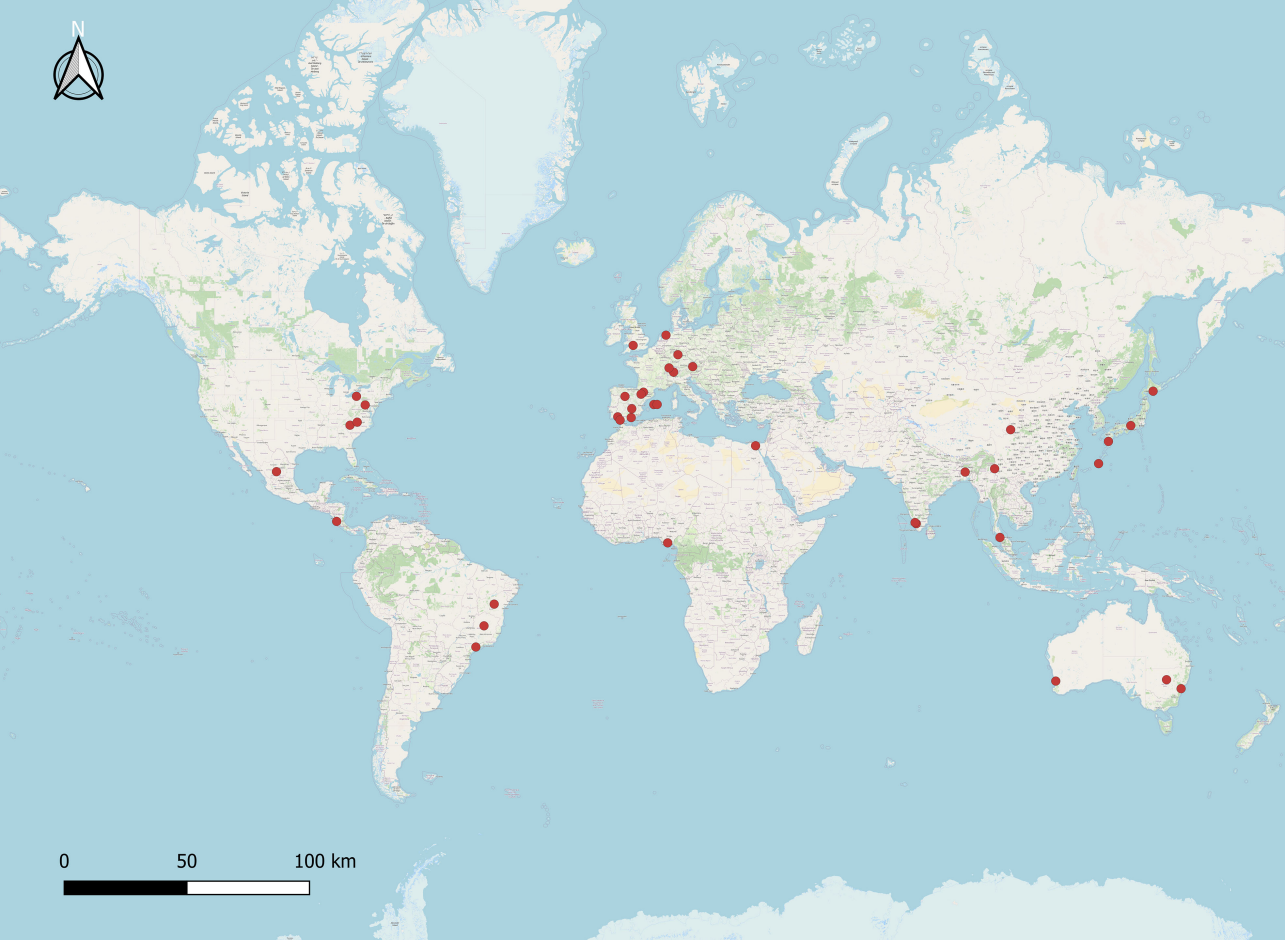
Global records on ant-mediated pollination studies. QGIS-employed ant pollination study sites (red dots).

**Figure 2:**
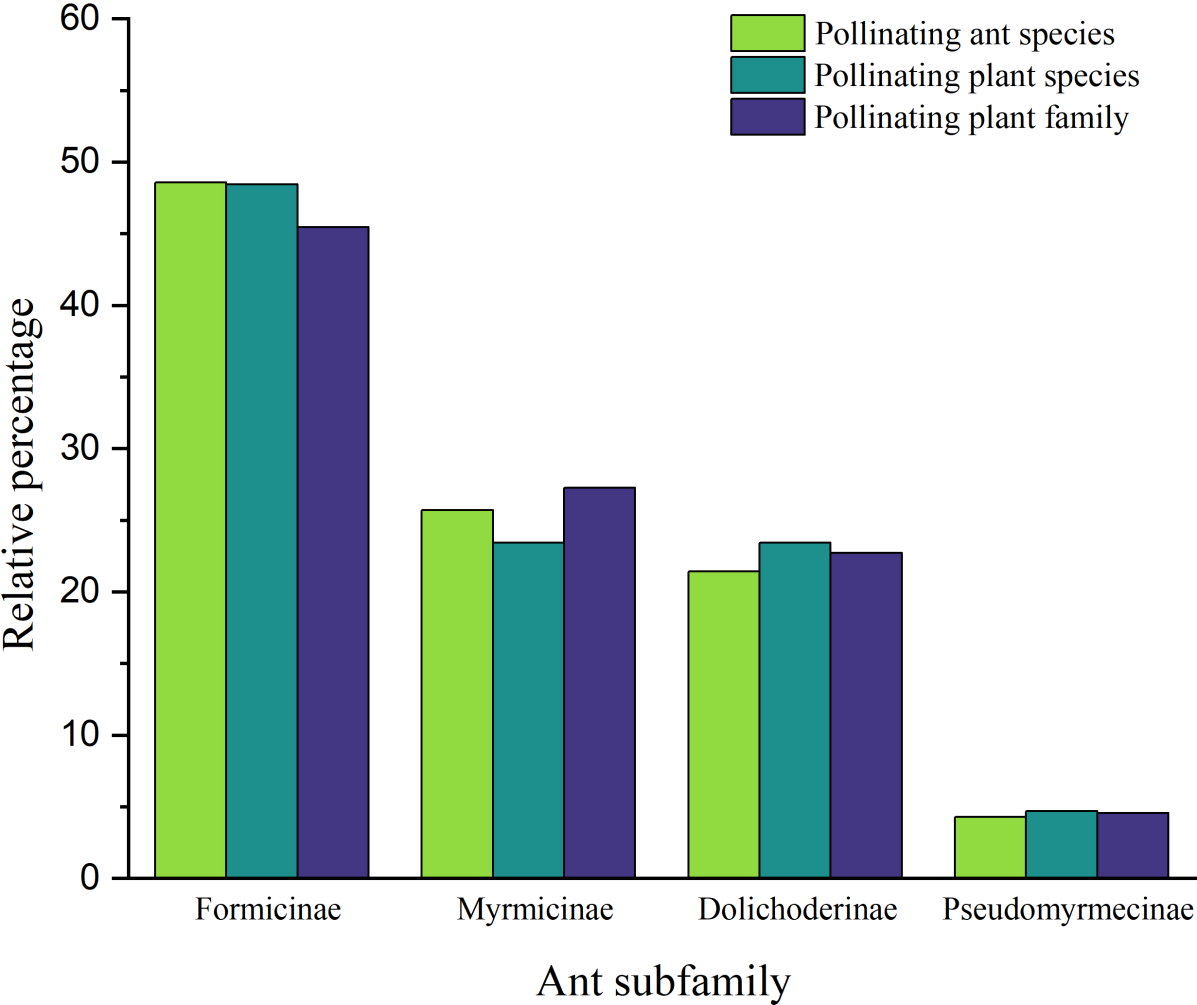
Overall distribution of the relative proportion of pollinating ants (species) and plants (species and family) from the studied ant subfamilies.

The diversity of pollinating ants attending to their encountered plant(s), flower characteristics, and dispersion (**Figure 1**) (**Table 2 Supplementary File 1**) was prepared based on the previously published information (**Table 1**). A total of 70 ant species (Hymenoptera: Formicidae) from four subfamilies (Formicinae, Myrmicinae, Dolichoderinae, and Pseudomyrmecinae) were identified as pollinators of 41 plants from 23 families based on the data compiled from published records. Among the ant subfamilies, Formicinae accounts for the largest share of pollination (species, n = 34; 48.571%) based on the number of species sampled, and it was observed that those in the Myrmicinae (n = 18; 25.714%) and Dolichoderinae (n = 15; 21.428%) subfamilies pollinated with a lower intensity than Formicinae. The role of Pseudomyrmecinae ants in pollination is less established (n = 3; 4.285%). There was a significant variation of pollinating ants across subfamilies (χ2 = 2.794; df = 3; p = 0.000) (**Figure 2**). Furthermore, it was observed that Formicinae ants pollinated a wide range of flowers (pollinating plant species, n = 31), accounting for about 48.437% of all pollinated plants. Records for Myrmicinae (pollinating plant species, n = 15) and Dolichoderinae (pollinating plant species, n = 15) were equally substantial, indicating their comparable plant-tending behaviors (both pollinated 23.437% of all pollinated plants); however, they varied in terms of the diversity of pollinated flowers. Pseudomyrmecinae ants were observed to pollinate only a few plant species (n = 3; 4.687% of all pollinating plants). There was a highly significant difference in myrmecophilous pollination among the ant subfamilies for different plant species (χ2 = 2.475; df = 3; p = 0.000) (**Figure 2**). Although only 41 plant species belonging to 23 families were documented to have any myrmecophilous pollination, some plants were pollinated by more than one ant species (**Table 1**).

**Figure 3:**
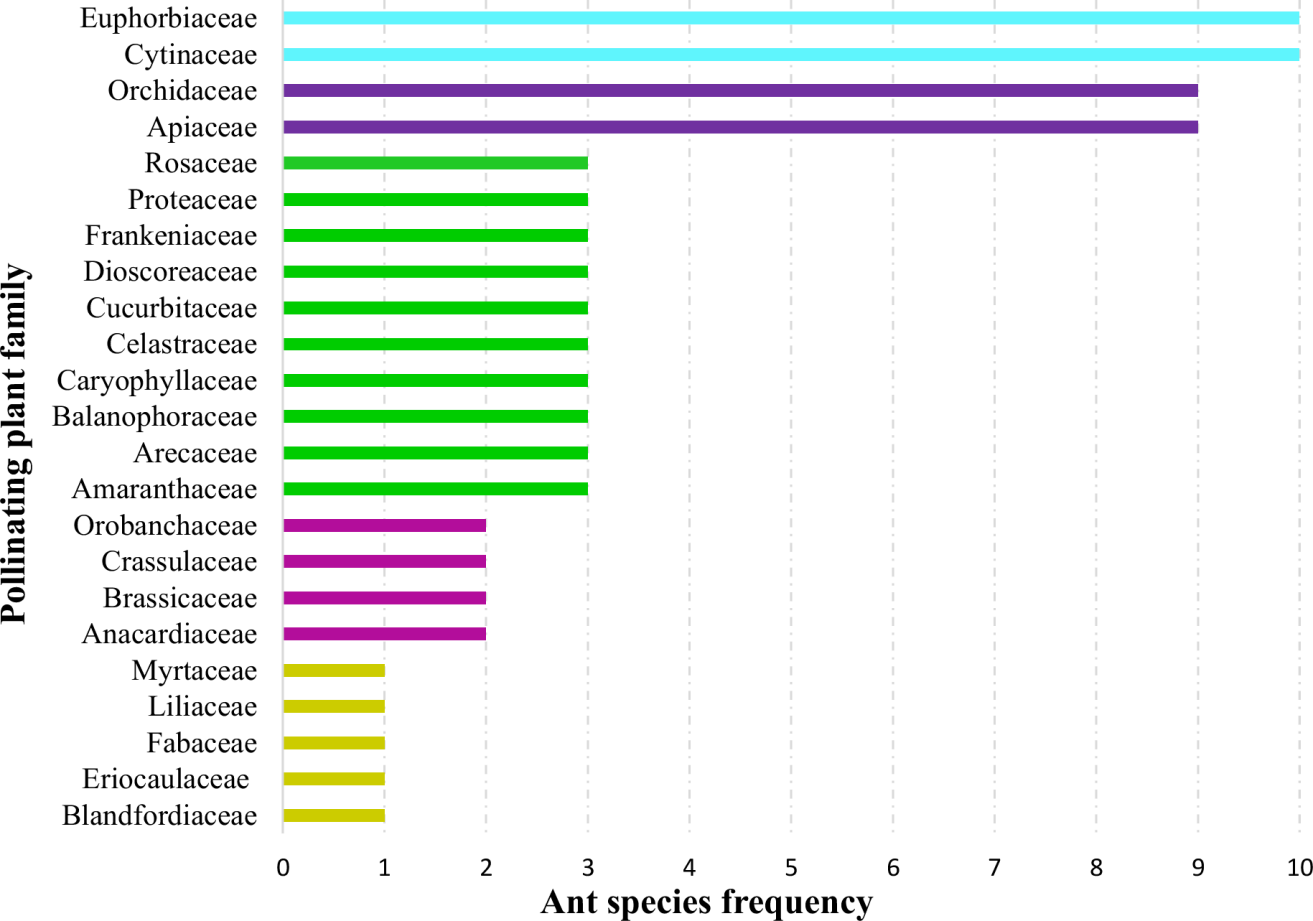
Distribution of pollinating ants to plant families. Each bar represents how many ant species pollinate a specific plant family. The same-color bars represent an equal number of ant species (species may vary) pollinating different plant families.

**Figure 4:**
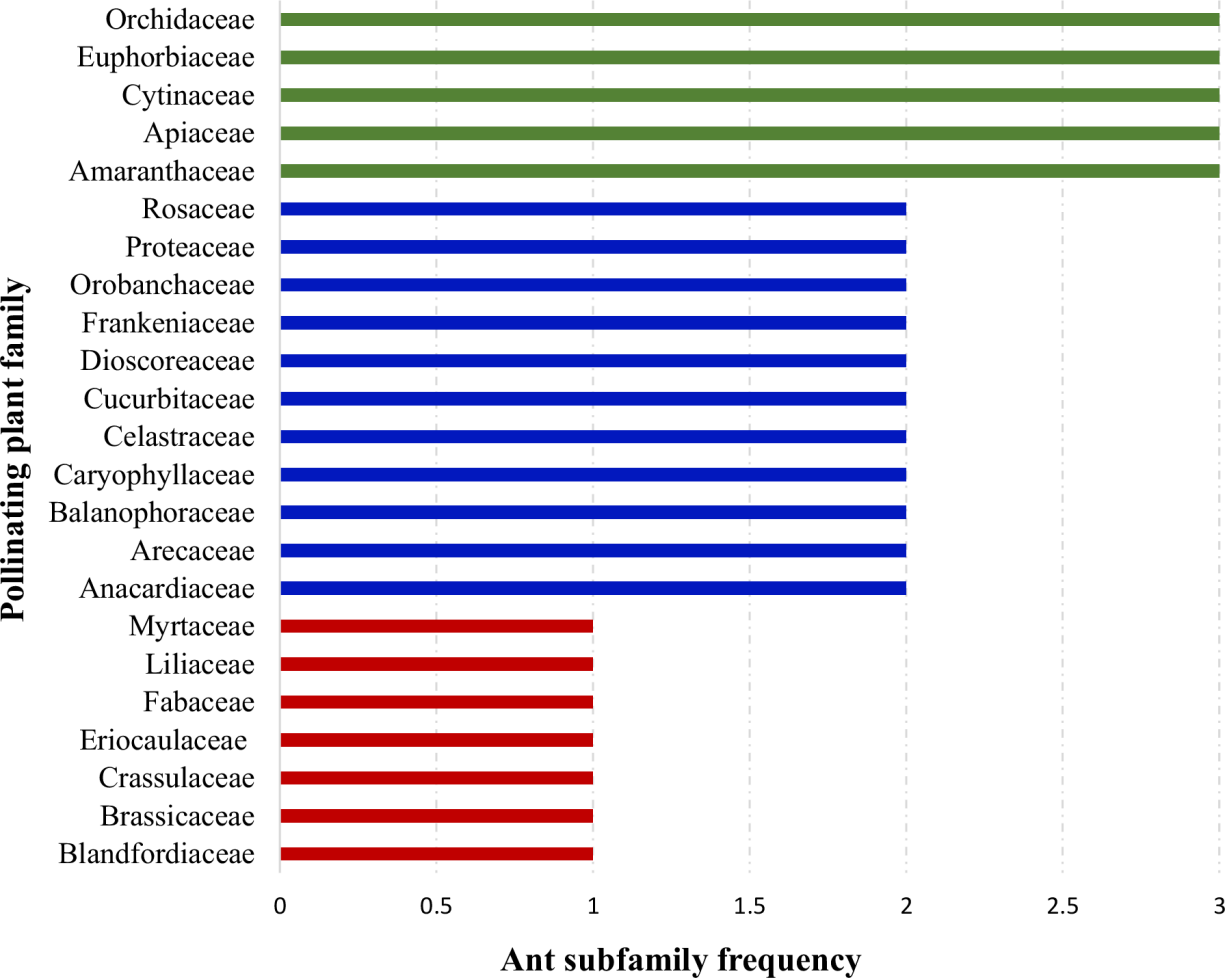
Distribution of pollinating ants to plant families. Each bar represents how many ant subfamilies pollinate a specific plant family. The same-color bars represent the equal number of ant subfamilies pollinating different plant families.

**Figure 5:**
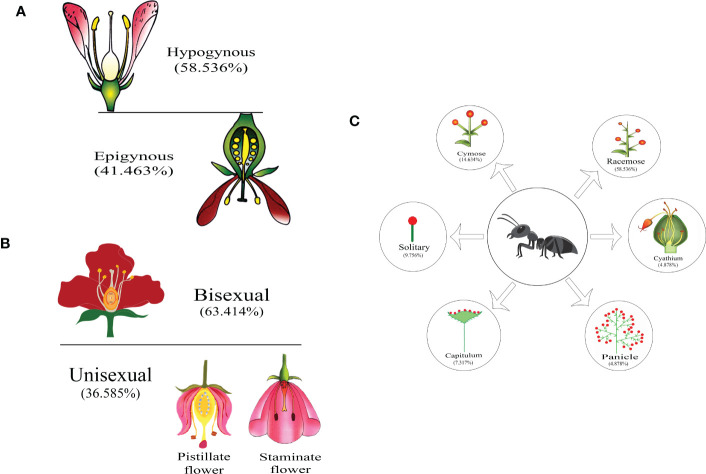
Myrmecophily according to floral characteristics: **(A)** ant pollination to flower ovary position, **(B)** ant pollination to flower sex, and **(C)** ant pollination to flower inflorescence. The magnitude of pollination is displayed in percentages for each case.

When plant families were considered, we found that Formicinae-pollinated plant families were the most common (n = 20; 45.454%), followed by those pollinated by Myrmicinae (n = 12; 27.272%), Dolichoderinae (n = 10; 22.727%), and Pseudomyrmecinae (n = 2; 4.545%). The result shows a statistically significant difference (χ2 = 1.491; df = 3; p = 0.001) (**Figure 2**) (**Table 1**). At least five plant families (Blandfordiaceae, Eriocaulaceae, Fabaceae, Liliaceae, and Myrtaceae) were exclusively pollinated by a single (but not the same) species of ant. Records showed that only 9 or 10 ant species have been recorded as pollinating the Apiaceae, Orchidaceae, Cytinaceae, and Euphorbiaceae families. However, only two or three ant species were reported as being possible pollinators of 14 different plant families (**Figure 3**) (**Table 1**). A significant difference was observed in the number of ant species with their respective pollinating plant families (χ2 = 5.331; df = 22; p = 0.000). This indicates that plant families are exclusive enough for ants to accomplish pollination. Furthermore, at least seven different plant families (Blandfordiaceae, Brassicaceae, Crassulaceae, Eriocaulaceae, Fabaceae, Liliaceae, and Myrtaceae) were observed to be exclusively pollinated by ants from a single subfamily. At least 11 plant families (Anacardiaceae, Arecaceae, Balanophoraceae, Caryophyllaceae, Celastraceae, Cucurbitaceae, Dioscoreaceae, Frankeniaceae, Orobanchaceae, Proteaceae, and Rosaceae) were observed to be exclusively pollinated by any two subfamilies, whereas at least five plant families (namely Amaranthaceae, Apiaceae, Cytinaceae, Euphorbiaceae, and Orchidaceae) were observed to be pollinated by any three ant subfamilies (**Figure 4**) (**Table 1**). However, there was no significant variation among the ant subfamilies that pollinated the plant families (χ2 = 2.434; df=2; p= 0.002). Although the majority of ant-pollinated flowers were hypogynous (n = 24; 58.536%) rather than epigynous (n = 17; 41.463%), no significant difference in results was observed (χ2 = 1.195; df = 1; p = 0.002) (**Figure 5A**) (**Table 1**). This indicates that angiosperms with floral parts, such as sepals, petals, and stamens, attached either to the upper part of the ovary (hence less exposed) or the lower part of the ovary (hence highly exposed), do not have any significant difference in pollination. We found that a larger proportion of bisexual flowering plants was pollinated (n = 26; 63.414%) than unisexual flowering plants (n = 15; 36.585%) (χ2 = 2.951; df = 1; p = 0.008) (**Figure 5B**) (**Table 1**). **This** suggests that floral sexuality may have a modest effect on pollination. In terms of inflorescence, racemose (n = 24; 58.536%), cymose (n = 6; 14.634%), and solitary (n = 4; 9.756%) flowers were the most commonly pollinated, and capitulum (n = 3; 7.317%), cyathium (n = 2; 4.878%), and panicle (n = 2; 4.878%) flowers were occasionally pollinated. When pollination success on floral inflorescences was statistically analyzed, significant variations were found (χ2 = 5.339; df = 5; p = 0.000) (**Figure 5C**). This finding suggests that flowering plant petal configurations significantly influence ant pollination.

Out of the 70 pollinating ants, 56 pollinate to only one plant species, 10 pollinate to no more than two, three pollinate to no more than three, and only one pollinates to no more than four. On the other hand, among all pollinated plant species (n = 41), 19 plants were pollinated by only a single ant species, nine plants were pollinated by a maximum of any two, and nine other plants were pollinated by a maximum of any three. However, three different plant species were shown to be pollinated by four, five, or six distinct ant species. Only one plant was found to have been pollinated by up to 10 different ant species. According to the records, only Formicinae ants pollinated five plant families (Blandfordiaceae, Brassicaceae, Crassulaceae, Eriocaulaceae, and Fabaceae). Myrmicinae and Dolichoderinae were the only pollinators of at least two plant families (Liliaceae and Myrtaceae). According to the available evidence, Formicinae is both a generalist and specialist pollinator. For example, Formicinae with Pseudomyrmecinae may co-pollinate two plant families (Arecaceae and Celastraceae); Formicinae and Dolichoderinae may co-pollinate three plant families (Proteaceae, Caryophyllaceae, and Rosaceae); Formicinae and Myrmicinae may co-pollinate six plant families (Anacardiaceae, Balanophoraceae, Cucurbitaceae, Dioscoreaceae, Frankeniaceae, and Orobanchaceae); and again, Formicinae, along with Myrmicinae, and Dolichoderinae may co-pollinate five plant families (Amaranthaceae, Apiaceae, Cytinaceae, Euphorbiaceae, and Orchidaceae). The results showed that Formicinae ants were responsible for pollinating the most plant families (n = 20), followed by Myrmicinae (n = 12), Dolichoderinae (n = 10), and Pseudomyrmecinae (n = 2).

**Figure 6:**
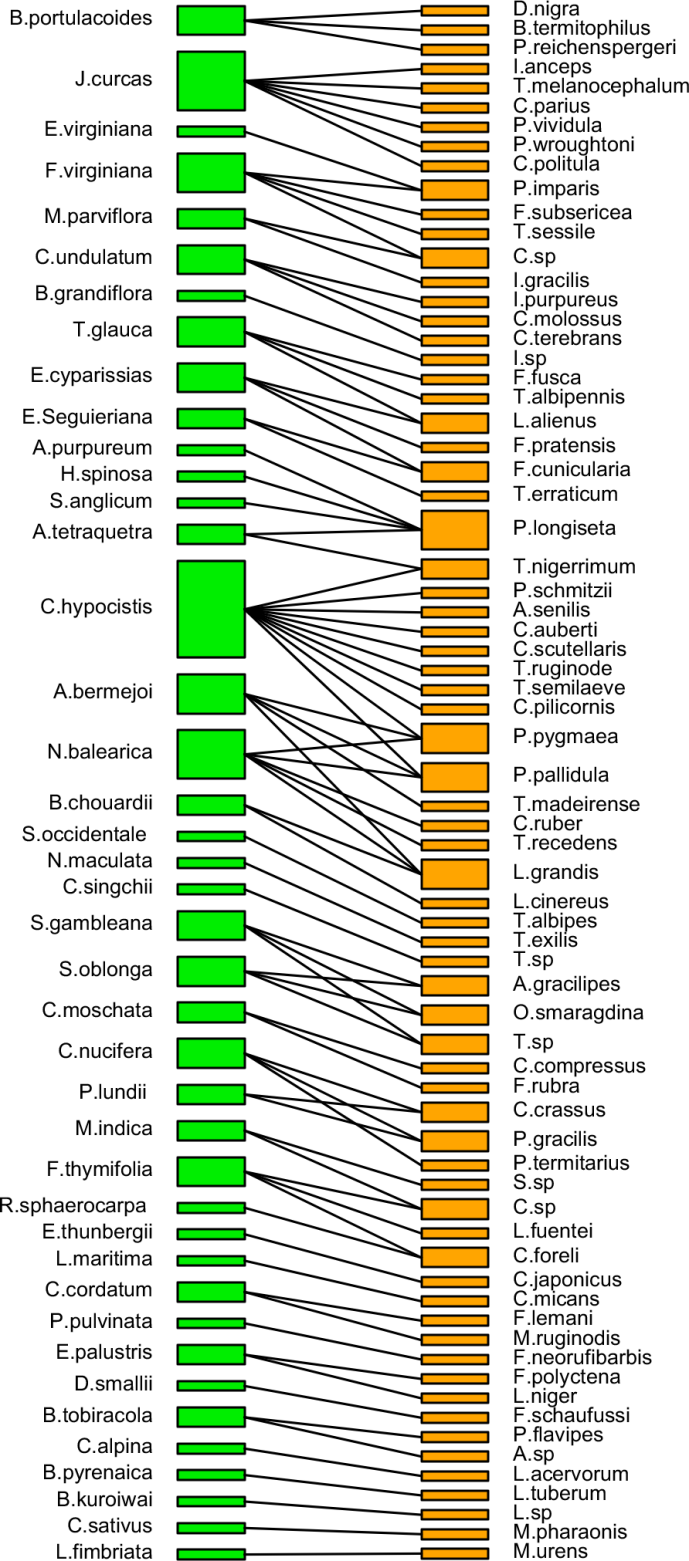
A bipartite network connecting pollinating ant species (upper orange nodes) and pollinated plant species (lower green nodes). The widths of the nodes represent the marginal total of pollinations made by a pollinator species or received by a plant species. There are 70 distinct ants and present 41 different plant species, displaying an interaction network based on the likelihood of pollination between the groups, which is indicated by the black line linking single or branched lines between two nodes. Thick upper nodes indicate the presence of the ant species with the highest pollination activity, whereas thin nodes indicate the presence of the ant species with the lowest (or least) pollination activity. Thick lower nodes indicate the presence of the most-pollinated plant species, whereas thin nodes indicate the presence of least-pollinated plant species.

**Figure 7:**
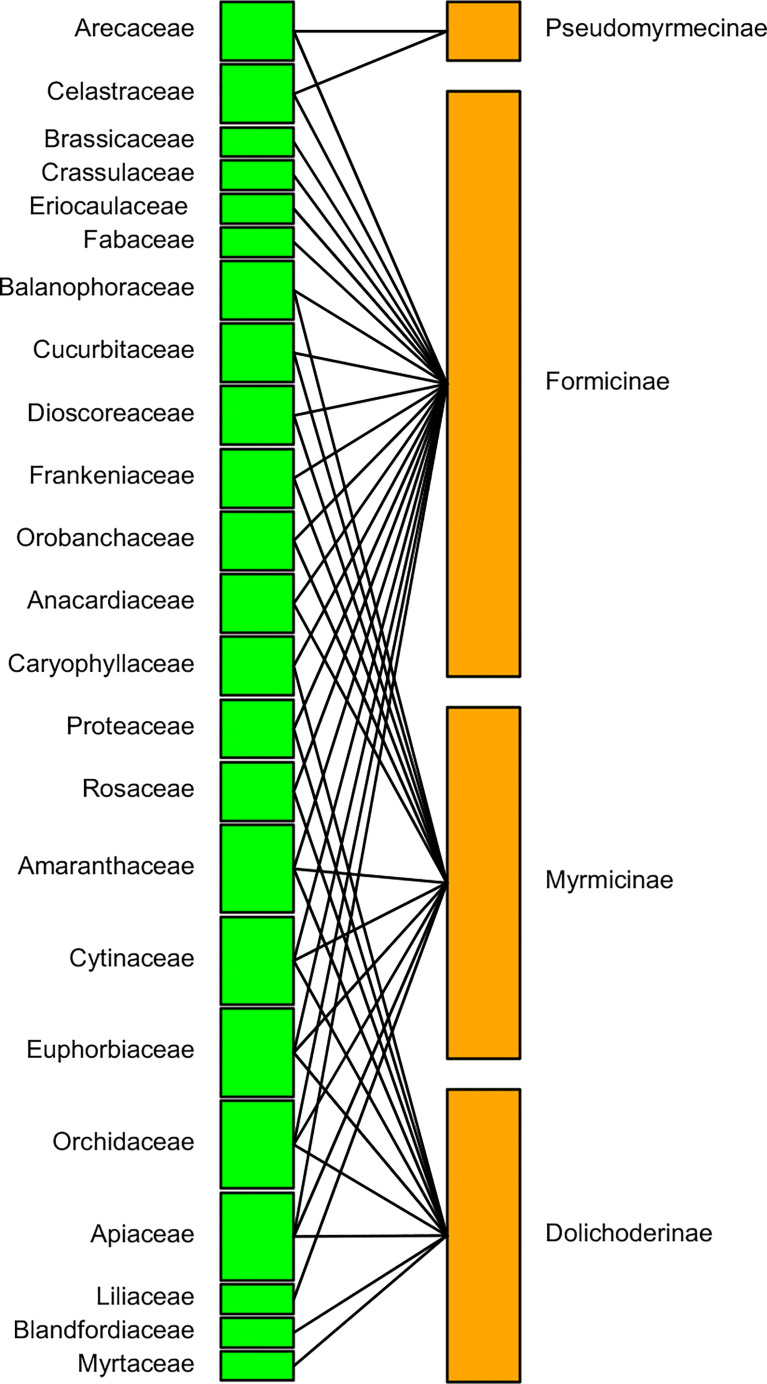
A bipartite network connecting pollinating ant subfamilies (upper orange nodes) to pollinated plant families (lower green nodes). The node widths show the marginal total of pollinations made by pollinator ant subfamilies or received by plant families. There are four ant subfamilies and 23 plant families, with an interaction network depending on the likelihood of pollination between the groups, which is denoted by a single or branched black line connecting two nodes. Thick upper nodes indicate the presence of the most ant subfamilies, whereas thin nodes indicate the presence of the fewer ant subfamilies. Thick lower nodes indicate the presence of most-pollinated plant families, whereas thin nodes indicate the presence of least-pollinated plant families.

The pollinating ant–plant bipartite networks are shown in **Figures 6**, **7**. The analysis of the bipartite interaction network between pollinating ants (upper orange nodes) and their various pollinating plants (lower green nodes) made it clear that the networks were significantly less nested (NODF = 1.797). It suggests that the network is unstable enough to account for the likelihood of substantial plant-to-ant linkages. Furthermore, the extent of specialization in this bipartite network was more significant than the average (H2′ = 0.898), indicating the existence of a specialized network between pollinating ants and the plant species they visited (**Figure 6**) (**Table 2, Supplementary File 2**). On the other hand, the bipartite network between distinct pollinating ant subfamilies (upper orange nodes) and pollinating plant families (lower green nodes) was found to be significantly nested (NODF = 55.682). This implies that the network comprises several generalist pollinating ant subfamilies interacting with different plant families. The degree of specialization (H2′ = 0.394) in such a bipartite network demonstrated the robustness of pollination interaction (**Figure 7**) (**Table 2, Supplementary File 3**).

**Figure 8:**
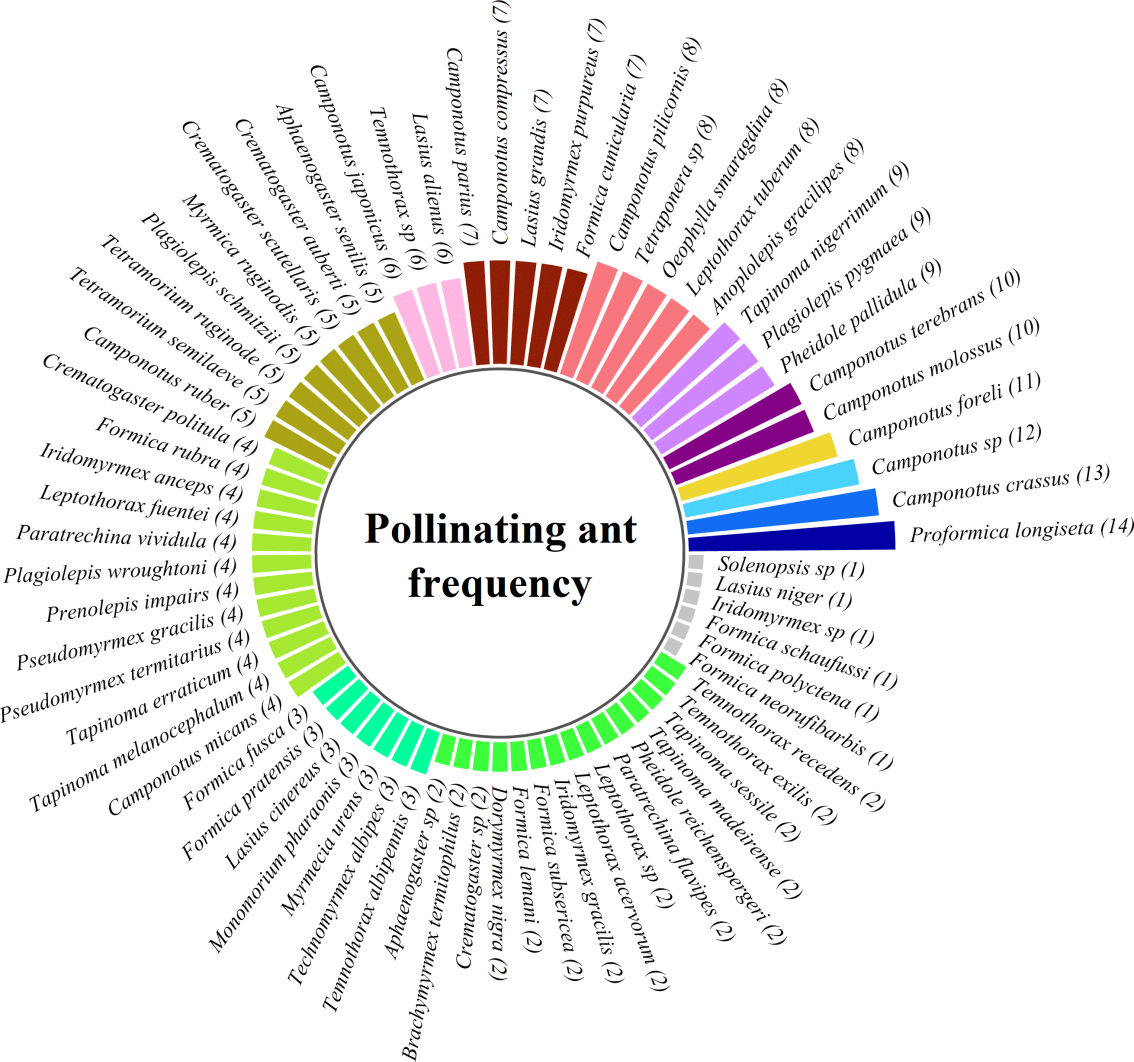
The pollinating ant frequency according to available records. The number after an ant species reflects how many times the species was studied in different publications by different authors. For example, “*Proformica longiseta* (14)” means that 14 different publications (by different authors/author groups; multiple studies on an ant species in different times by the same author/author group were ignored) recorded *Proformica longiseta* pollinating plants (same or different plant species).

**Figure 9:**
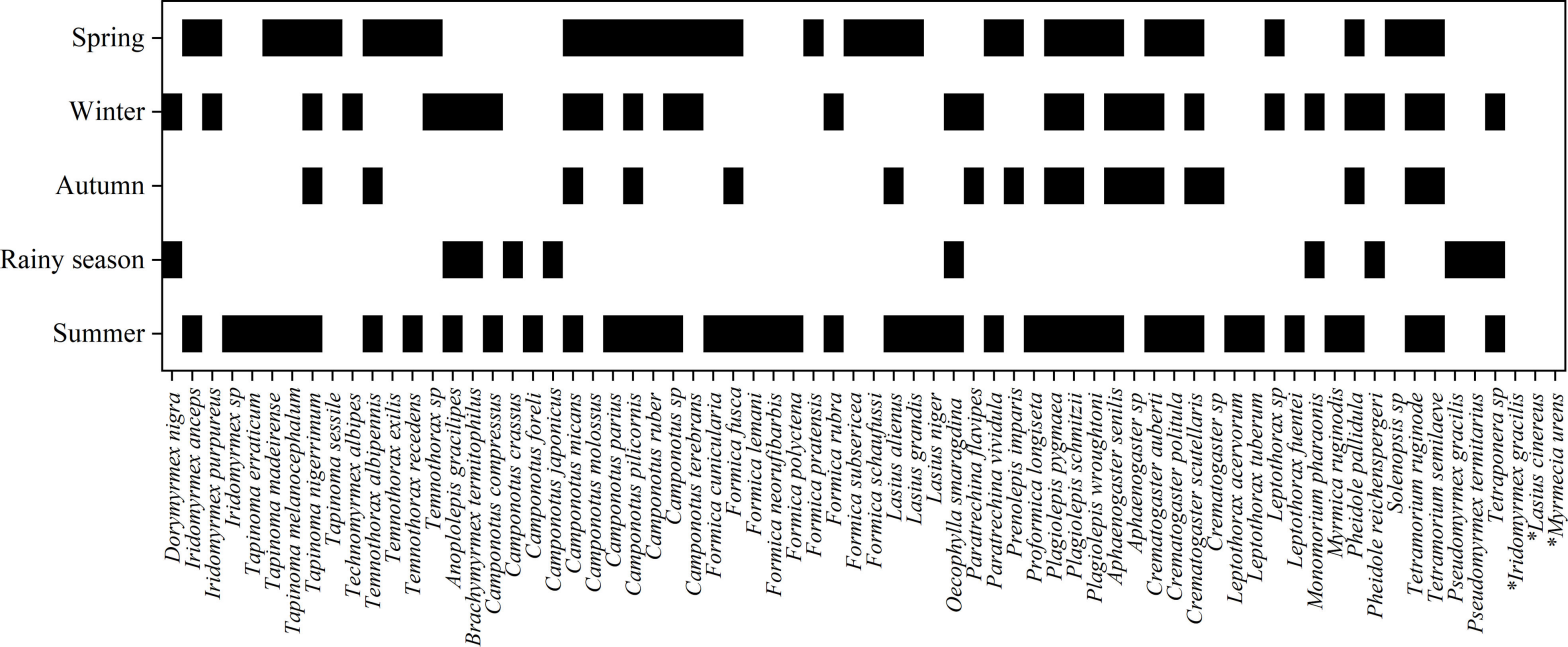
Ant pollination according to the seasons. The heat map illustrates the pollinating ant(s) with their pollination season(s). The black bars indicate pollination; the white bars indicate no pollination. Seasonal indications are missing in literature records for the final three species (marked with asterisks).

According to the studied publications, pollination frequency on a single plant species or multiple plant species indicates that the endemic polygynous ant *Proformica longiseta*, which lives in the high mountains of Europe, is the most frequent ant-pollinator. Their pollination ability was most documented (on 14 occasions). Likewise, 11 publications have confirmed that *Camponotus crassus*, which lives in bromeliads, bamboo, and the cocoa plantations of The Cerrado, South America, is the second most frequent pollinator. At least seven different species of ants were reported to pollinate by only a single publication (**Figure 8**) (**Table 2, Supplementary File 4**). **Figure 8** summarizes the number of publications reporting sightings of individual ant species engaged in pollination (numbers appear after ant names). Ant pollination performance varied over the year, which may be related to the success of their pollen dispersal efforts. According to our data, ants pollinated more frequently in the summer (case studies, n = 43; 30.935%) and spring (n = 38; 27.338%) than in the winter (n = 29; 20.863%) and fall (n = 18; 12.949%), but less frequently in the rainy season (n = 11; 7.913%). However, there was a statistically significant difference in ant pollination across seasons (χ2 = 2.571; df = 4; p = 0.000) (**Figure 9**) (**Table 2, Supplementary File 5**).

## Discussion

The current review delves into the intricacies of ant pollination and explores data on how ants from different subfamilies perform this service for plants. Our findings reveal that when species-specific links are included, the pollination interaction network between ants and plants is less stable than when only the specificity of interactions between ant subfamilies and plant families is considered. Therefore, more generalized networks were observed at higher taxonomic levels (i.e., subfamilies or above), and more specialized networks emerged at lower taxonomic levels (i.e., species). Network ecology, in terms of stability and specialization among the involved species, has most likely been optimized in ant-to-plant pollination events over time, underscoring the vital relevance of co-evolution among the participants. It has been demonstrated that myrmecophilous ant–plant associations influence the dynamic nature of network structural integrity across various arthropod groups (95, 96). Herbivorous insects have highly specialized systems because they feed on specific plant species (97), but predatory arthropods can alternate between insect prey and floral rewards (98). Arthropods typically have a very stable and robust interaction matrix as a result of their extensive resource utilization (i.e., extensively layered network design) (99); however, in agricultural contexts, plant–pollinator networks are frequently considered as being specialized rather than nested (100). The Formicinae subfamily has the most species diversity, presumably allowing Formicinae ants to pollinate one or several plants and successfully boost pollination probability. They also exhibit higher levels of behavioral efficiency than most other groups because of their powerful mouthparts and flexible bodies, although this efficiency is not always independent of environmental stability. They are inundated with nectar during peak flowering periods, allowing them to pollinate a wide variety of flowering plants (48, 101). The presence of nectar increases the frequency of ant visits to flowers, thereby increasing the chances of pollination (101, 102). Moreover, Formicinae ants often visit the reproductive structures of foliages in quest of floral and extra-floral nectars, resulting in increased pollination. Pollination is facilitated by the visitor’s foraging skill and frequency (38, 42, 54, 101). Ants with superior foraging abilities are likely to regularly travel extensively on numerous panicles, flower sepals, and petals. For example, *Camponotus* ants visit floral structures more frequently than others, allowing them to pollinate more successfully (34). Thus, both shorter and more extended foraging periods may increase pollination, although longer foraging intervals may result in more pollen removal and retention on stigmas (63, 103), increasing the possibility of cross-pollination and seed germination (65). The morphology of the pollinating species, in addition to the plant parts, is essential to and promotes pollination. Ants, particularly those belonging to the genus *Camponotus*, have thick, hairy bodies that facilitate the passage of pollen; hence, plants may rely on ants as pollen transfer vectors, a process known as geitonogamy. *Camponotus terebrans* are efficient pollinators as they have physical characteristics that allow them to pollinate various flowers (57). Even when the North American winter ants *Prenolepis impairs* and *Crematogaster* sp. are present in the same flower, *P. impairs* is capable of pollinating plants more effectively due to its morphological advantages and integumental architectures (35, 72, 77). The Mediterranean ant *Plagiolepis pygmaea* is capable of adhering the anthers to its body, carrying different amounts of pollen by adhering pollen grains to its head, thorax, and gaster (35). Floral structures, particularly the openness of reproductive organs exhibited in chasmogamous flowers, greatly assist pollination (57). Ant-mediated cross-pollination occurs regularly in the chasmogamous flowers of two American beech species, *Fagus grandifolia* and *Epifagus virginiana*, because of their favorable floral features, such as their open and exposed anthers and stigmas (77).

According to myrmecophilous pollination statistics, ants from the Myrmicinae and Dolichoderinae subfamilies have less species diversity than the Formicinae subfamilies but more than the Pseudomyrmecinae subfamilies (63). As a result, Myrmicinae and Dolichoderinae were identified as the second and third most common pollinating ant subfamilies after Formicinae. Nonetheless, only a few Pseudomyrmecinae ant species have been recorded doing this job. Most ant species were found to be “generalist” pollinators, meaning that they pollinated a wide range of plants from various families, and only a few ant species exhibited “specialist” behavior and pollinated only a subset of flowering plants. Even if these results are skewed due to the content of the published sources we analyzed, Formicinae is projected to have a higher pollination frequency than others due to its greater abundance and vast distribution. Their superior pollination capacity indicates they can thrive in a broader range of environmental constraints than other subfamilies.

In contrast to Formicinae, Myrmicinae and Dolichoderinae ants interacted with a limited number of plants, indicating that they have specialized pollination hosts. Pseudomyrmecinae ants interact with plants in a more specialized manner, exhibiting the group’s more specialized pollination behavior (58, 60). Although inferring a linear relationship between an ant’s specialist or generalist behavior and the number of hosts encountered may be erroneous, we can reject the null hypothesis by assuming that, due to “positive complementarity” (104, 105), the chance of pollination is decreased when an ant visits only specific plants. Nonetheless, it appears that Myrmicinae, Dolichoderinae, and Pseudomyrmecinae ants have received less attention, resulting in fewer findings. Their poor ranking could be attributed to the fact that they might pollinate infrequently or ineffectively in the wild. Pollination deficiency in Myrmicinae and Dolichoderinae ants can be explained by their limited abundance in agricultural fields and woodlands, as the majority of the species appear to live in deserts and dunes. As a result of inefficient behavioral labor, Myrmicinae and Dolichoderinae are poor pollinators. Ants that visit plants for food are more apparent on vegetation, and are therefore capable of more effective pollination than ants that receive food through predation. Predatory Pseudomyrmecinae are rare pollinators because they prefer animal meat to plant-derived foods. They have been reported as more inclined to engage in hostile interactions with others to obtain sustenance through predation rather than gathering nectar, thereby reducing the likelihood of pollination.

Our investigation reveals that pollination success in plants varies across plant families. This could be due to beneficial plant features such as physical (color, odor, and texture) or chemical (foliar and extra-foliar nectar) cues that encourage the pollinators (69, 106–108). When ants consume nectar from flowers, they pollinate the plants without intending to do so because food resources, such as nectar, are natural ant attractants. According to our findings, ants frequently pollinate plants from four families (Euphorbiaceae, Cytinaceae, Orchidaceae, and Apiaceae) due to their appealing flower structure, color, appearance, and higher nectar production (35, 63, 66, 109). For example, ants are naturally drawn to the vibrant colors of Orchidaceae blooms (110, 111). Euphorbiaceae flowers are effectively pollinated due to their unusually elongated shapes and overall floral composition (67). The pollination interactions between ants and plants can be considered to involve negative trade-offs between species. However, such interactions are well-documented as exercises in mutualistic cooperation.

We evidenced that ants pollinate hypogynous flowers more than epigynous flowers (63). Since the ovary position is superior in hypogynous flowers, pollen rapidly clings to it, and the pollen easily moves from the stigma to the ovary *via* the stamen, resulting in pollination. As the ovary position is inferior in epigynous flowers, pollen transfer from the stigma to the ovary is primarily influenced by the transit distance, which is significantly shorter in hypogynous than in epigynous flowers. As a result, the ovary position of the flower is critical for insect pollination. As pollen has to travel further to reach an ovary with an inferior position, ants are assumed to be more likely to pollinate flowers with a superior ovary position. Therefore, they are assumed to have co-evolved more closely with hypogynous than epigynous flowers.

Flower sex also influences myrmecophilous pollination. According to our data, bisexual blooms exceed unisexual flowers in pollination success. As bisexual flowers have both male and female sexual organs, when ants visit these flowers they inadvertently move the distinct sexual parts of the blossoms, resulting in pollination. However, ants’ responses to male and female unisexual blooms are different. Pollinator visits to a unisexual flower do not ensure fertilization due to the separation of male and female reproductive organs. As a result, the presence of ants at unisexual flowering plants (facultative association) leads to a low rate of successful pollination (112, 113) compared with the rate for bisexual flowering plants (114).

According to our findings, one ant species may be able to pollinate as many as seven distinct types of flowering plants. The most effective pollination occurs in racemose flowers, where the blooms continuously develop along the rachis, and by traveling along the blossoms ants can pollinate efficiently. In addition to racemose, cymose inflorescences were also found to be highly pollinated by ants. Successful pollination depends on anther dehiscence (anther splitting). For instance, the longitudinal dehiscence seen in members of the Brassicaceae family allows the androecium to be fully or partially exposed and dispersed among the flowers. Solitary flowers, which have a single flower unit in the central axis, are visited by ants in the same way that racemose flowers are; however, the pollination rate is lower in solitary than in racemose flowers due to the singularity of the flowering unit. Flowers from cymose, cyathium, panicle, and capitulum inflorescences are pollinated by ants to a significantly lesser degree, likely due to their “defined” idiosyncratic flowering architectures. It has been recorded that some flower-visiting ants roam along the margins or borders of the petals but do not pollinate due to unappealing floral characteristics, including nectar (60, 114); however, if other floral features are present, ants pollinate quite efficiently (63, 65). Thus, floral morphology, particularly the number of flower components and flower structure, and animal morphology, especially ants’ exoskeletal armature, influence pollination success (115, 116, 117).

Although ant pollination occurs throughout the year, it is most widespread in the spring and summer due to ants’ increased foraging activity during the warmer months (67, 69, 70). They are frequently active in warmer regions in search of food and are therefore attracted to various flowers for pollen and nectar, ultimately pollinating them. For example, ants are highly active pollinators in Southeast Asia during the summer and spring when mango flowers extensively (65), but in the United States they are highly active during the fall, when *Epifagus virginiana* blooms profusely (77). However, they typically stop pollinating when it rains (36).

## Conclusion

There is often a high degree of specificity in the interactions between ants and plants during pollination, although floral patterns and seasons can lead to variability in this process. In contrast to the general mutualistic networks of pollinating ants and plants, antagonistic networks among members show partner-specific interactions. The dynamics of network evolution differ between mutualistic and antagonistic communities; partner-switching is more critical to the ebb and flow of interactions in the former than in the latter, but species turnover is far more critical in the latter. However, antagonistic and mutualistic networks underwent significant ecological and topological changes, with varying consequences for pollinator and herbivore populations in agricultural settings. This review supports the view that pollinators are more agile in their engagement choices than herbivores, which are more reciprocally specialized and fix unstable affiliations. Our findings demonstrate that predictive analyses of the overall plant–ant bipartite network in a given agro-environment are crucial for guiding diversity and analyzing its various ecological benefits. More studies on the subject across diverse agroecological systems will provide insight into the optimal complexity of habitats in degraded or intensified agricultural landscapes. It will provide new opportunities for identifying diverse ants, which may have a definitive role in pollination.

The cognitive processes behind ants’ pollination instincts are obscure. Despite their high intelligence, it is reasonable to consider the possibility that ants pollinate flowers both on purpose and by coincidence while gathering food. Hence, it is essential that an ethological inquiry is undertaken to reveal the cognitive processes employed by ants as they carry out pollination tasks.

## Author contributions

SD and AD conceived and designed the manuscript. SD collected and computed the data, analyzed the results, and graphically presented them. SD was primarily responsible for drafting the manuscript, and AD later revised and corrected it. Both authors finally discussed and verified the results and statistics. All authors contributed to the article and approved the submitted version.
